# Probiotic Supplementation Improves Glucose Homeostasis and Modulates Interleukin (IL)-21 and IL-22 Levels in Pediatric Patients with Type 1 Diabetes: A Randomized Placebo-Controlled Trial

**DOI:** 10.3390/metabo15050288

**Published:** 2025-04-24

**Authors:** Amira Abdel Moneam Adly, Eman Abdel Rahman Ismail, Mahasen Mohamed Abd-Elgawad, Nouran Yousef Salah

**Affiliations:** 1 Department of Pediatrics, Faculty of Medicine, Ain Shams University, Cairo 11591, Egypt; amiraadly73@med.asu.edu.eg (A.A.M.A.); nouranyousef@med.asu.edu.eg (N.Y.S.); 2 Department of Clinical Pathology, Faculty of Medicine, Ain Shams University, Cairo 11591, Egypt; 3 General Practitioner, Ministry of Health, Cairo 11591, Egypt; s.elnady@fci-cu.edu.eg

**Keywords:** probiotics, type 1 diabetes, glucose homeostasis, interleukin-21, interleukin-22

## Abstract

**Background:** Probiotics alter gut microbiota and have beneficial effects on immune homeostasis. The role of probiotics in diabetes has been shown in some studies. Interleukin (IL)-21 and IL-22 have been implicated in the pathogenesis of type 1 diabetes mellitus (T1DM). Objectives: This study aimed to assess the effect of oral supplementation with probiotics on glycemic control and IL-21 and IL-22 levels in pediatric patients with T1DM. **Methods:** This randomized controlled trial was registered in ClinicalTrials (NCT04579341) and included 70 children and adolescents with T1DM. They were randomly assigned into two groups to receive either an oral probiotic tablet containing 0.5 mg *Lactobacillus acidophilus* once daily or a matching placebo. Both groups were followed up for 6 months with assessment of fasting blood glucose (FBG), lipids, hemoglobin A1c (HbA1c), and IL-21 and IL-22 levels. **Results:** Baseline clinical characteristics and laboratory parameters were similar between both groups (*p* > 0.05). After six months, probiotic supplementation for the intervention group resulted in significant decreases in FBG, HbA1c, total cholesterol, and IL-21 levels, while IL-22 was increased compared with baseline levels (*p* < 0.001) and compared with the placebo group (*p* < 0.001). No adverse reactions were reported. Baseline IL-21 was positively correlated to FBG, HbA1c, and total cholesterol while there were negative correlations between these variables and IL-22 levels. **Conclusions:** Probiotic supplementation improved glucose homeostasis and glycemic control, possibly through their immunomodulatory effects on cytokines IL-21 and IL-22. Thus, probiotics could be a safe adjuvant therapy to intensive insulin in pediatric patients with T1DM.

## 1. Introduction

The role of gut microbiota as an important determinant of health in humans has emerged in the past decade [1,2]. Gut microbiota alterations are thought to be implicated in the pathogenesis of metabolic disorders including diabetes, obesity, and cardiovascular disease. Failure of preservation of the mucosal barrier’s integrity may cause systemic endotoxemia, which contributes to chronic low-grade inflammation and leads to the development of metabolic syndrome [3]. Moreover, gut microbiota play a crucial role in the pathogenesis of type 1 diabetes mellitus (T1DM) [4,5].

Probiotics are living micro-organisms that, when supplied in adequate amounts, confer health benefits to the host [6,7]. Probiotics have been used to ameliorate gastrointestinal manifestations. They have been thought to influence gut microbiota, their polysaccharide antigens, and their key metabolites [8]. Moreover, probiotics were found to improve epithelial barrier function by increasing adhesion protein levels, including E-cadherin and beta-catenin [9]. In addition, probiotics have immune-protective influence on the host through stimulation, regulation, and modulation of the immune responses. Probiotics also have an immunomodulatory effect through influencing cytokine production by antigen-presenting cells, which initiates adaptive responses [10].

Owing to their immunomodulatory role, probiotics have been widely used in many autoimmune, inflammatory, and allergic diseases. Probiotics were found to lower blood glucose and insulin levels in diabetes in preclinical settings as well as clinical trials in humans. However, these studies differ from each other in the species of probiotics used, their dose, and their magnitude of efficacy [11]. Studying the immunomodulatory effect of probiotics and their potential role in the prevention or alleviation of certain pathologies for which proper medical treatment is as yet unavailable has gained attention. Moreover, isolation of new probiotic strains and investigating their immunomodulatory effects on cytokines in humans remain topical issues [10].

Mucosal homeostasis confers physical and molecular interactions between gut microbiota, the epithelial layer, and the local immune system. Interleukin (IL)-22 is a cytokine produced by gut immune cells and helps to orchestrate this three-way interaction [12]. It plays a crucial role in antimicrobial immunity and the integrity of the intestinal mucosal barrier. IL-22 has diverse metabolic benefits; it preserves the gut mucosal barrier, ameliorates insulin sensitivity, decreases systemic inflammation and endotoxemia, and regulates lipid metabolism [3]. Exogenous IL-22 reduced the endoplasmic reticulum and oxidative stress in human pancreatic islet cells and enhanced insulin secretion from beta cells [13,14].

Interleukin (IL)-21 is a type 1 multifunctional cytokine that has been involved in the pathogenesis of several autoimmune diseases, including T1DM [15]. It is produced by activated T lymphocytes, particularly the inflammatory Th17 subset, and is believed to have a key role in the interaction between innate and acquired immunity. It has also been related to several inflammatory and autoimmune diseases [16]. IL-21 production by T cells has been shown to be increased in children with newly diagnosed T1DM and in adults with established T1DM [17,18]. Moreover, IL-21 enhances the production of intestinal immunoglobulin A (IgA) against microbiota [19]. We hypothesized that probiotics might influence the levels of cytokines (IL-21 and IL-22), and thus may have beneficial effects on glucose homeostasis. This study aimed to assess the effect of probiotic supplementation on glycemic control and blood glucose levels as well as the levels of IL-21 and IL-22 in pediatric patients with T1DM.

## 2. Materials and Methods

This was a prospective randomized, double-blinded, placebo-controlled study. It was approved by the local ethical committee (FMASU MS 5/2025) and registered in ClinicalTrials.gov with the registration number (NCT04579341). Seventy children and adolescents with T1DM were recruited from the regular attendees of the Pediatric and Adolescents Diabetes Unit, Pediatric Hospital. Informed consent was taken from each participant or their legal guardians prior to the study. The reporting of the study conforms to the Consolidated Standards of Reporting Trials 2010 statement [20].

Patients with T1DM according to the criteria of the American Diabetes Association (ADA) [21] on regular insulin therapy, aged 5–18 years, with at least 5-year diabetes duration and without micro-vascular complications were recruited. Exclusion criteria were clinical evidence of infection, obesity, the presence of comorbid medical conditions (i.e., celiac disease or autoimmune thyroiditis), history of liver or renal disease, previous administration of any vitamins or food supplements 1 month before enrollment, and involvement in any investigational trial within 3 months prior to the study.

### 2.1. Sample Size

Sample size was calculated using the STATA program, setting the type-1 error (α) at 0.05 and the power (1-β) at 0.8. Results from a previous study [22] showed that the mean change (decrease) in HbA1c after probiotic supplementation was (−1.21), while there was a 0.2 mean increase in hemoglobin A1c (HbA1c) among the control group, with an assumed 1.5 common standard deviation. Calculation according to these values produced a sample size of 35 cases per group after taking into consideration a 10% dropout rate.

### 2.2. Randomization and Study Population

**Figure 1:**
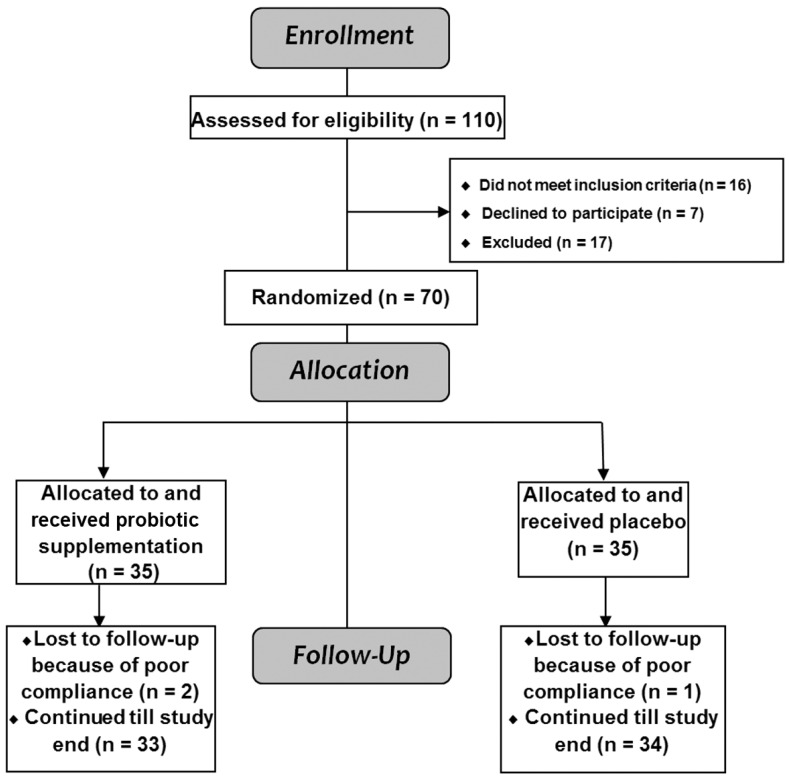
CONSORT flow diagram for the enrolled patients with type 1 diabetes mellitus.

A total of 110 patients with T1DM were screened for eligibility; 16 patients did not meet the inclusion criteria, 7 patients declined to participate, 17 patients were excluded, and 70 patients were enrolled (Figure 1). Probiotics were administered according to a predetermined protocol generated from random numbers in a 1:1 manner according to a computer-generated randomization sequence preserved within the investigational drug pharmacy, with allocation concealment by opaque sequentially numbered sealed envelopes. Patients and investigators were blinded to treatment during the study through the use of visually identical tablets and packaging for the trial medication and placebo. The 70 patients were randomly assigned into one of two groups (35 patients in each group) to receive either oral probiotic tablets (Acidophilus Probiotic, Nature’s Bounty, Bohemia, NY, USA) containing 0.5 mg *Lactobacillus acidophilus* La-14 (10^8^ colony-forming units [CFU]) in a dose of one tablet per day or a matching placebo. The placebo was composed of sunflower soft gelatin tablets that appear identical to the probiotic tablets and was prepared by Morgan Chemical Industries Co., El Sharkeya, Egypt.

All patients with T1DM were treated with a basal–bolus regimen that included subcutaneous insulin Glargine (Lantus^®^; Sanofi-Aventis, Frankfurt, Germany) as the basal insulin and insulin Aspart (NovoRapid^®^, Novo Nordisk, Copenhagen, Denmark) as the mealtime insulin, with a mean dose of 1.2 ± 0.25 IU/kg/day. Insulin doses were adjusted according to the International Society for Pediatric and Adolescent Diabetes (ISPAD) guidelines [23] during the monthly visits.

### 2.3. Baseline Clinical Assessment and Laboratory Investigations

Baseline detailed medical history was taken, focusing on diabetes duration, history of severe hypoglycemia or diabetic ketoacidosis (DKA) with number of hospital admissions, and history of chronic micro- and macro-vascular complications. Anthropometric measures were assessed by calculation of the standard deviation score (SDS), and blood pressure was measured by comparison of values to normal reference percentiles [24].

Mean fasting blood glucose (FBG) was assessed in the last three months prior to the study, routine liver and kidney function tests were conducted using a Beckman Coulter AU480 chemistry analyzer (Beckman Coulter Electronics, Hialeah, FL, USA), and mean HbA1c% in the last year prior to the study was assessed using D-10 (BioRad, Marnes La Coquette, France). Serum levels of human IL-21 and IL-22 were assessed by an enzyme-linked immunosorbent assay (ELISA) with the use of ELISA kits from Bioassay Technology Laboratory, Shanghai Korain Biotech Co., Ltd. (Shanghai, China).

### 2.4. Follow-Up

Probiotics were administered for six months to show a clear effect on glycemic control and levels of IL-21 and IL-22. All patients were closely and clinically followed up every four weeks to assess treatment compliance and monitor any signs of potential adverse effects during the study period. Adherence to treatment was assessed based on pill counts of dispensed and returned medication, and patients who received less than 80% of the study medication were considered non-compliant [25]. All participants were instructed to maintain their usual diet and physical activity habits throughout the study period and to refrain from substantial changes in their lifestyle habits. Simultaneously with pharmacological treatment, nutrient intake and physical activity were assessed through 24 h dietary recall, which was carried out by a pediatric dietician through interviews with the enrolled patients or their caregivers during each visit.

### 2.5. Statistical Analysis

Data were analyzed using the Statistical Package for Social Science (IBM SPSS) version 27. A Kolmogorov–Smirnov test was used to examine the normality of data. Variables were presented as means and SD or median and interquartile range (IQR; 75th and 25th percentiles). Differences between the intervention and placebo groups as regards age, disease duration, and percent change of the studied variables were assessed using the independent sample Student’s t-test for quantitative parametric data, while the Mann–Whitey test was used for non-parametric data. Categorical parameters were compared by the chi-square test. To determine the effects of probiotics on body mass index (BMI), blood pressure, insulin dose, and laboratory variables, an analysis of covariance (ANCOVA) was performed. Variables which were not normally distributed were log-transformed before entering the analysis. The within-group changes (before and after 6 months of intervention) were evaluated using the paired-samples t-tests or Wilcoxon rank-sum test. Spearman correlation coefficients and multivariable linear regression analysis were used to assess the relation between IL-21 as well as IL-22 levels and other studied variables. A *p*-value < 0.05 was considered significant in all analyses. The analyses were conducted based on the intention-to-treat principle.

## 3. Results

### 3.1. Baseline Clinical and Laboratory Data of the Enrolled Participants with T1DM

A total of 70 children and adolescents with T1DM were enrolled, namely 38 males and 32 females, with a mean age of 8.5 ± 2.4 years (range: 5–14 years). Patients were randomly assigned, with 35 each in the intervention and placebo groups. Both groups were followed up for six months. No significant difference was found between baseline clinical and laboratory data among T1DM patients with and without probiotic supplementation (Table 1), and none of the patients were hypertensive or obese. A total of 3 patients dropped out of the study because they did not attend follow-up measurements; 2 of these participants were in the probiotics group and 1 was in the placebo group, leaving 67 patients for the intention-to-treat analysis.

### 3.2. Effect of Probiotic Supplementation on Glycemic Control, Lipid Profile, and IL-21 and IL-22 Levels

As shown in Table 1, patients with T1DM who received probiotic supplementation had significantly lower systolic and diastolic blood pressure, FBG, RBG, HbA1c, triglycerides, and total cholesterol compared with baseline levels or the placebo group. Moreover, IL-21 levels were markedly decreased, while IL-22 was significantly higher at the end of follow-up compared with baseline levels and the control group. However, no significant difference was found between the baseline and the study end as regards any of the studied clinical or laboratory data among patients with T1DM who did not receive probiotics. No adverse reactions to oral supplementation with probiotics were recorded throughout the six months of therapy.

### 3.3. Correlation Between Baseline ILs and Other Studied Variables Among T1DM Patients

**Figure 2:**
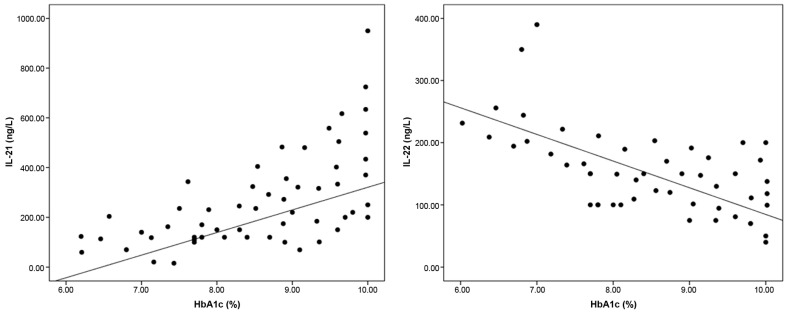
Correlation between baseline HbA1c and each of IL-21 and IL-22 levels in patients with type 1 diabetes mellitus.

There were significant positive correlations between baseline IL-21 levels and each of FBG (r = 0.445, *p* < 0.001), HbA1c (r = 0.566, *p* < 0.001), and total cholesterol (r = 0.375, *p* < 0.001), while IL-22 was negatively correlated with diastolic blood pressure (r = −0.248, *p* = 0.039), FBG (r = −0.367, *p* = 0.026), HbA1c (r = −0.451, *p* < 0.001), and total cholesterol (r = −279, *p* = 0.031) (Figure 2). IL-21 and IL-22 were negatively correlated with each other (r = −0.603, *p* < 0.001). Multivariable linear regression analysis (Table 2) showed that FBG, HbA1c, and total cholesterol were the significant independent variables that affect IL-21 and IL-22 levels among T1DM patients.

## 4. Discussion

Studies reported significant differences in the relative abundance of different gut microbiota, including *Lactobacillus*, *Bacteroides*, *Clostridium cluster XIVa*, *Prevotella*, and *Bifidobacterium*, between healthy individuals and patients with T1DM. This dysbiosis has been shown to increase intestinal permeability and thus promotes the development of a pro-inflammatory niche which stimulates an autoimmune mechanism in beta cells among susceptible individuals [26].

Animal studies assessed the role of manipulating gut microbiota to modulate the onset of T1DM in predisposed subjects. Transplantation of gut microbiota from MyD88-deficient non-obese diabetic mice, which are protected from T1DM development, into NOD mice resulted in a significant reduction in the onset of insulitis and a delay in diabetes onset [27].

The role of microbiota in the pathogenesis of diabetes is well established. However, previous evidence regarding the anti-diabetic properties of probiotics is based mainly on animal studies, without enough human studies [6], particularly in patients with T1DM. A meta-analysis by Tonucci et al. [28] showed conflicting results about the beneficial effect of probiotics in people with type 2 diabetes mellitus (T2DM), with only few studies evaluating the possible underlying cause, including markers of oxidative stress, inflammation, and incretins. Therefore, further studies are needed to assess the effects of probiotics on glycemic control in T1DM and the underlying mechanisms involved in this complex relationship [28].

Administration of genetically modified strains of *Lactobacillus lactis* that secrete pro-insulin autoantigen and the immunomodulatory cytokine IL-10 could revert diabetes in NOD mice and increase the frequencies of local T regulatory cells [29]. Moreover, administration of *Lactobacillus lactis* that expresses glutamic acid decarboxylase (GAD)-65 and IL-10, combined with short-course low-dose anti-CD3, was found to stabilize insulitis, preserve functional β-cell mass, and restore normoglycemia in NOD mice with recent-onset diabetes [30]. A study involving Japanese patients with T2DM showed that probiotics decreased bacterial translocation and gut dysbiosis [31].

In this study, probiotic supplementation for six months led to declines in blood glucose levels and improved glycemic control and dyslipidemia in pediatric patients with T1DM, without adverse effects. In line with these results, several animal and human studies suggested anti-diabetic properties for *Lactobacillus* and *Bifidobacteria* [32,33]. Oral administration of *Lactobacillus johnsonii* strain La1 for two weeks restored normoglycemia following an oral glucose load in streptozotocin-induced diabetic rats [34].

Similarly, Calcinaro et al. [35] found that early oral administration of VSL#3, which is a probiotic mixture containing *S. thermophiles*, *Bifidobacterium*, and *Lactobacillus*, prevented diabetes development, reduced insulinitis, and decreased β-cell destruction in NOD mice. In agreement with these data, Dolpady et al. [36] showed that treatment with oral probiotic VSL#3, alone or combined with retinoic acid, had a protective role for NOD mice from T1DM. They suggested that this approach acts by decreasing the inflammasome at the intestinal level through inhibition of IL-1β and enhancement of the release of protolerogenic components, such as IL-33 and indoleamine 2,3-dioxygenase. Harisa et al. [37] also showed that administration of *Lactobacillus acidophilus*, whether alone or combined with acarbose, significantly decreased FBG, HbA1c, and malondialdehyde (MDA) levels in diabetic rats. In concordance with these results, Honda et al. [38] demonstrated that *Lactobacillus rhamnosus* GG administration (108 CFU/mL) for six weeks resulted in improving glycemia through enhancing insulin sensitivity, adiponectin production, and increasing glucose transporter type 4 (GLUT4) expression in diabetic mice [39].

Furthermore, Ostadrahimi et al. [22] found that supplementation with probiotic fermented milk (kefir) reduced FBG and HbA1C among patients with T2DM compared with controls. Fasting triglycerides, total cholesterol, and LDL cholesterol were also decreased compared to baseline data; however, these changes were not statistically significant [22].

The glycemic regulatory effect of probiotics could be due to stimulation of gut microbiota to produce glucagon-like peptide-l and insulinotropic polypeptides which in turn increase the uptake of glucose by the liver and muscles [22,40]. Additionally, this anti-diabetic effect may be attributed to the antioxidant effect of probiotics by several interacting mechanisms, which eventually lead to glycemic regulation [22]. Moreover, probiotics improve intestinal barrier integrity and decrease pathogenic micro-organisms and their toxic end products such as lipopolysaccharide (LPS). A reduction in LPS leads to a reduction in circulating pro-inflammatory cytokines that are involved in insulin resistance in patients with T2DM [41,42]. Thus, probiotics, through their anti-inflammatory properties [35,43], can help reduce inflammation and regulate glucose and lipid metabolism in people with diabetes.

The current study suggests that the protective role of probiotics among people with T1DM is most likely related to modulation of IL-21 and IL-22 levels, as shown by the improvement in their levels after probiotic supplementation; patients with T1DM who received probiotic supplementation had markedly decreased IL-21 and significantly higher IL-22 at the end of the follow-up compared with baseline levels or the placebo. Moreover, FBG and HbA1c were significant independent variables affecting IL-21 and IL-22 levels. To the best of our knowledge, previous studies addressing the effect of probiotics on IL-21 and Il-22 are lacking.

In this context, Sutherland et al. [15] investigated the role of IL-21 in T1DM. They generated IL-21R-deficient NOD mice and C57Bl/6 mice expressing IL-21 in pancreatic β cells to determine the effect of IL-21 deficiency and excess on T1DM. IL-21R expression deficiency was found to render NOD mice resistant to insulitis, formation of insulin autoantibodies, and T1DM onset. Conversely, overexpression of IL-21 was found to induce inflammatory modulators, including IL-17F, IL-17A, monocyte chemoattractant protein (MCP)-1, MCP-2, interferon (IFN)-γ, and IFN-inducible protein-10, in the pancreas. This led to leukocytic infiltration in the pancreatic islets, causing β-cell destruction and T1DM development in the normally diabetes-resistant C57Bl/6 NOD mice. The authors attributed the disease-promoting activity of IL-21 to recruitment of CD4+ cells and macrophages to inflamed islets [15]. These data are consistent with other studies [44,45] which showed that IL-21R−/−NOD mice are protected from insulitis and T1DM.

These findings highlighted the prodiabetogenic activity of IL-21 on diverse genetic backgrounds, which suggests that the use of IL-21 antibodies, blocking agents, or IL-21R-Fc fusion proteins could be a promising therapeutic target for the prevention or treatment of T1DM [15].

On the other hand, interleukin (IL)-22 is an anti-inflammatory cytokine that has a protective role against islet cell inflammation and glucose-induced toxicity [46]. Abadpour et al. [46] investigated the effect of IL-22 on human islets exposed to both hyperglycemia and LIGHT. They found attenuation of the upregulation of LIGHT receptors (LTβR and HVEM) that occurred in engrafted human islets exposed to hyperglycemia by IL-22. The authors suggested that the harmful effect of hyperglycemia and LIGHT on pancreatic islet function was through enhancement of endoplasmic reticulum stress, as evidenced by increased secretion of pro-inflammatory cytokines and the release of pro-coagulant mediator tissue factor, which led to pancreatic islet cell apoptosis, and all of this was counteracted by IL-22 which promoted islet cell function and survival.

Interestingly, patients who received probiotics showed significant decreases in triglycerides and total cholesterol at the end of therapy compared with their baseline levels or the placebo. Various studies have shown that probiotic supplementation, especially with *Lactobacillus* and *Bofidobacteria*, can reduce serum cholesterol levels. Thus, these probiotics can be useful in the management of dyslipidemia [3,47].

In line with our study, *L. acidophilus* and *B. lactis* probiotic administration in people with T2DM was found to improve total cholesterol and low-density lipoprotein (LDL) cholesterol. Probiotic supplementation was suggested to be beneficial in controlling the cardiovascular risk factors of diabetes [6]. In another study, although probiotic supplementation in people with T2DM had no effect on FBG, total cholesterol, triglycerides, or LDL cholesterol compared to the placebo group, it increased high-density lipoprotein (HDL) cholesterol levels [48].

In Iran, Ejtahed et al. [6] found that consumption of probiotics (*Lactobacillus acidophilus* La5 and *Bifidobacterium lactis* Bb12) for six weeks improved HbA1c, FBG, and the antioxidant status of people with T2DM. Probiotics were found to significantly increase glutathione peroxidase and erythrocyte superoxide dismutase activities and the total antioxidant status (TAS) compared with controls.

Ostadrahimi et al. [22] suggest that the hypocholesterolemic properties of probiotics could be due to the use of cholesterol by probiotics for their own metabolism. They assume that binding of probiotics to cholesterol converts it to a catabolic product. Furthermore, probiotics decrease cholesterol levels by deconjugating cholesterol to bile acids. In addition, *L. acidophilus* was found to inhibit 3-hydroxy 3-methyl glutamyl CoA reductase, an enzyme responsible for endogenous cholesterol synthesis, eventually reducing total body cholesterol concentration [49].

Limitations of this study include the lack of direct measurement of gut microbiota changes by stool analyses or 16S ribosomal RNA (16S rRNA) sequencing to confirm that *Lactobacillus acidophilus* successfully colonized or altered participants’ microbiomes. Moreover, this study did not evaluate the long-term effect of probiotics after their discontinuation to determine whether the beneficial effects of probiotics on glycemic control are sustained after the cessation of therapy. Larger prospective trials with extended follow-up are needed to assess the long-term effects of probiotic supplementation.

## 5. Conclusions

In conclusion, we suggest that oral probiotic supplementation in the form of 0.5 mg *Lactobacillus acidophilus* La-14 (108 CFU) once daily for six months improved glycemic and lipid profiles in children and adolescents with T1DM without any adverse reactions. Probiotics possibly exerted their actions on glucose homeostasis through their immunomodulatory effects on cytokines, as reflected by lowering IL-21 and increased IL-22 levels. Thus, probiotics could be a safe adjuvant therapy to intensive insulin in pediatric patients with T1DM. Further larger studies with longer duration of probiotic supplementation are needed to clarify which probiotic strains and doses are most effective in controlling T1DM, as well as their effect on diabetic vascular complications. Investigating other immunological markers such as IL-10, tumor necrosis factor-α, T-regulatory cells, or IgA secretion represents a promising area for future research for better characterization of the immunomodulatory mechanisms by which probiotics could influence glycemic control in diabetes.

## Figures and Tables

**Table 1:** Clinical and laboratory data of patients with type 1 diabetes mellitus receiving probiotics therapy or placebo at baseline and at 6 months.

Variable	Probiotics				Placebo				*p*-Value ^b^
	Baseline (n = 35)	At 6 Months (n = 33)	Percent Change	*p*-Value ^a^	Baseline (n = 35)	At 6 Months (n = 34)	Percent Change	*p*-Value ^a^	
Age (years)	8.3 ± 2.6	-	-	-	8.7 ± 2.1	-	-	-	0.449 *
Males, n (%)	21 (60.0)	-	-	-	17 (48.6)	-	-	-	0.337 †
Disease duration (years)	6.8 ± 3.3	-	-	-	6.8 ± 3.1	-	-	-	0.941 *
BMI SDS	0.32 (−1.96–1.01)	0.53 (−1.76–1.21)	22.43 ± 12.16	0.152	0.29 (−1.13–0.93)	0.7 (−0.04–1.16)	25.31 ± 11.57	0.317	0.066
Systolic BP percentile	75.4 ± 11.3	67.2 ± 9.7	−11.2 ± 5.1	0.002	77.1 ± 10.2	81.4 ± 12.1	5.7 ± 1.3	0.124	<0.001
Diastolic BP percentile	80.2 ± 12.6	72.6 ± 10.7	−9.5 ± 3.2	0.008	81.1 ± 12.1	84.8 ± 13.2	4.2 ± 1.1	0.235	0.001
Insulin dose (IU/Kg/day)	1.18 ± 0.21	1.12 ± 0.11	−2.9 ± 2.1	0.194	1.21 ± 0.25	1.27 ± 0.3	0.9 ± 0.3	0.481	0.258
FBG (mg/dL)	170.5 ± 31.9	111.8 ± 17.9	−33.1 ± 12.6	<0.001	177.1 ± 31.2	182.4 ± 33.3	4.8 ± 2.8	0.393	<0.001
RBG (mg/dL)	313.3 ± 72.9	226.2 ± 41.1	−25.5 ± 15.6	<0.001	308.6 ± 34.7	313.1 ± 41.3	1.66 ± 1.3	0.384	0.001
HbA1c (%) HbA1c (mmol/mol)	9.12 ± 1.6 75.3 ± 9.7	7.73 ± 1.44 60.8 ± 8.1	−13.5 ± 8.7 −18.5 ± 9.8	<0.001 <0.001	9.09 ± 1.6 77.6 ± 8.9	9.26 ± 1.66 79.3 ± 9.9	3.4 ± 1.8 2.9 ± 1.0	0.569 0.316	<0.001 <0.001
Triglycerides (mg/dL)	0.50 ± 0.07	0.48 ± 0.07	−2.5 ± 2.0	0.205	0.49 ± 0.07	0.46 ± 0.09	−3.3 ± 2.6	0.200	0.340
Total cholesterol (mg/dL)	72.0 ± 17.6	61.9 ± 16.0	−3.9 ± 3.45	<0.001	73.4 ± 11.4	74.9 ± 10.5	1.8 ± 0.33	0.338	0.067
HDL cholesterol (mg/dL)	119.5 ± 27.1	95.9 ± 13.0	−15.8 ± 22.5	<0.001	111.8 ± 15.5	116.0 ± 13.4	5.6 ± 2.9	0.081	0.001
LDL cholesterol (mg/dL)	52.7 ± 13.2	59.23 ± 11.39	23.9 ± 11.0	0.056	55.6 ± 11.4	54.1 ± 14.2	−0.01 ± 13.8	0.825	0.186
IL 21 (ng/L)	180 (150–250)	50 (50–100)	−61.4 ± 25.8	<0.001	170 (120–220)	190 (170–250)	50.2 ± 21.7	0.075	<0.001
IL 22 (ng/L)	108 (70–150)	250 (200–375)	236.0 ± 62.4	<0.001	120 (100–170)	102 (57–152)	−7.03 ± 4.5	0.219	<0.001

SDS: standard deviation score; BMI: body mass index; BP: blood pressure; FBG: fasting blood glucose; HbA1c: hemoglobin A1c; HDL cholesterol: high-density lipoprotein cholesterol; LDL cholesterol: low-density lipoprotein cholesterol; IL-21: interleukin-21; IL-22: interleukin-22. ^a^ *p*-value was obtained from paired-samples t-tests for parametric variables or Wilcoxon rank-sum test for non-parametric variables. ^b^ *p*-value was obtained using analysis of covariance (ANCOVA) unless specified (*: Independent t-test was used; †: chi-square test was applied).

**Table 2:** Multivariable linear regression analysis for independent variables affecting baseline IL-21 and IL-22 levels in patients with type 1 diabetes mellitus.

Dependent Variable	Independent Variables	Unstandardized Coefficients		Standardized Coefficients	*p*-Value
		B	Standard Error	Beta	
IL-21	FBG (mg/dL)	1.097	0.300	0.384	0.002
	HbA1c (%)	24.386	4.195	0.303	0.011
	Total cholesterol (mg/dL)	1.253	0.987	0.249	0.009
IL-22	Diastolic BP percentile	−29.409	20.418	−0.158	0.155
	FBG (mg/dL)	−0.573	0.227	−0.28	0.014
	HbA1c (%)	−6.791	0.619	−0.265	0.026
	Total cholesterol (mg/dL)	−0.691	0.321	−0.239	0.035

IL-21: interleukin-21; IL-22: interleukin-22; FBG: fasting blood glucose; HbA1c: hemoglobin A1c; BP: blood pressure.

## Data Availability

Data will be made available upon request to the corresponding author.
